# A Dual-Mode Surface Acoustic Wave Delay Line for the Detection of Ice on 64°-Rotated Y-Cut Lithium Niobate

**DOI:** 10.3390/s24072292

**Published:** 2024-04-04

**Authors:** Philipp Schulmeyer, Manfred Weihnacht, Hagen Schmidt

**Affiliations:** 1 Leibniz Institute for Solid State and Materials Research, 01069 Dresden, Germany; p.schulmeyer@ifw-dresden.de; 2 innoXacs, 01744 Dippoldiswalde, Germany; innoxacs@online.de

**Keywords:** surface acoustic wave (SAW), SH-LSAW, Rayleigh wave, ice, lithium niobate, piezoelectric, sensor, finite-element method (FEM), non-destructive testing (NDT)

## Abstract

Ice accumulation on infrastructure poses severe safety risks and economic losses, necessitating effective detection and monitoring solutions. This study introduces a novel approach employing surface acoustic wave (SAW) sensors, known for their small size, wireless operation, energy self-sufficiency, and retrofit capability. Utilizing a SAW dual-mode delay line device on a 64°-rotated Y-cut lithium niobate substrate, we demonstrate a solution for combined ice detection and temperature measurement. In addition to the shear-horizontal polarized leaky SAW, our findings reveal an electrically excitable Rayleigh-type wave in the X+90° direction on the same cut. Experimental results in a temperature chamber confirm capability for reliable differentiation between liquid water and ice loading and simultaneous temperature measurements. This research presents a promising advancement in addressing safety concerns and economic losses associated with ice accretion.

## 1. Introduction

The accumulation of ice on infrastructure and machines compromises their performance and reliability, leading to safety concerns and economic losses. This applies to various industrial fields concerned with road, rail track and power line surveillance, wind power and aviation as well as with environmental and condition monitoring and instruments providing machine vision for unmanned vehicles. To address these issues, the development of robust and effective ice detection systems is crucial. Such systems not only allow the detection of iced surfaces but also serve as an essential prerequisite for de-icing procedures and provide indicators for the malfunction of anti- and de-icing systems. The purpose of this paper is to show a new non-destructive surface guided wave test approach in combined ice detection and temperature measurement by theoretical and experimental investigations using a surface acoustic wave (SAW) dual-mode delay line. Its significance is underlined by the technology’s benefits of small sensor designs, passive and wireless operation, retrofit options and capabilities for mass production [1]. The findings also strongly relate to investigations of liquid characteristics like required for lab-on-a-chip, biomedical or chemical process applications [2].

The concept of a SAW-based sensor for ice detection was first introduced by [3] using a delay line device deploying a shear-horizontal polarized leaky surface acoustic wave (SH-LSAW) on 36°-rotated Y-cut LiTaO_3_ (36LT) with propagation the along X direction. It was followed by the first devices deploying a Love wave in a SiO_2_ waveguide layer on ST-X+90° quartz [4] and 31°-rotated Y-cut quartz [5], respectively. Today’s aspirations in further developing SAW ice sensors still cover Love wave [6] as well as acoustic plate mode devices [7,8]. Moreover, dual-mode delay line devices are proposed by [9], consisting of a ZnO/quartz structure making use of the difference of Rayleigh-type wave’s dominating sagittal polarization strongly coupling with liquids for actuation (mixing) and the Love wave’s horizontal polarization weakly coupling with liquids for sensing purposes. Another dual-mode approach consisting of two separate acoustic propagation paths perpendicular to each other is proposed deploying Rayleigh-type waves and SH-SAW on 36LT for the determination of the acoustic properties of liquids [10].

In contrast, the work presented here demonstrates how Rayleigh-type wave and SH-LSAW utilized together in a dual-mode delay line (DM-DL) device on 64°-rotated Y-cut lithium niobate (64° YX LiNbO_3_, 64LN) with an electromechanical coupling coefficient approximately twice as large compared to 36LT can be used for ice detection. While recent investigations deploying SH-modes on 64LN deal with protein detection [11], hydrogen sulfide [12] or hydrogen sensing [13] as well as with microfluidic actuators [14] the choice of this substrate here has a different motivation. Due to its strong electromechanical coupling, 64LN supports efficient excitation of different modes providing capabilities for a wireless sensor device able to reliably differentiate between liquid water and ice loading and simultaneously measuring temperature.

## 2. Modal Analysis

64°-rotated Y-cut LiNbO_3_ is well known for its shear-horizontal polarized leaky SAW (SH-LSAW) with propagation along the crystallographic *X*-axis. A modal analysis is performed to find electrically excitable acoustic surface modes exhibiting a dominant polarization within the sagittal plane spanned by the propagation direction and the surface-normal direction. These can be either a Rayleigh-type wave (RW) with pure sagittal polarization or a generalized SAW (GSAW) with a significant sagittal polarization accompanied by a less dominant horizontal component.

**Figure 1:**
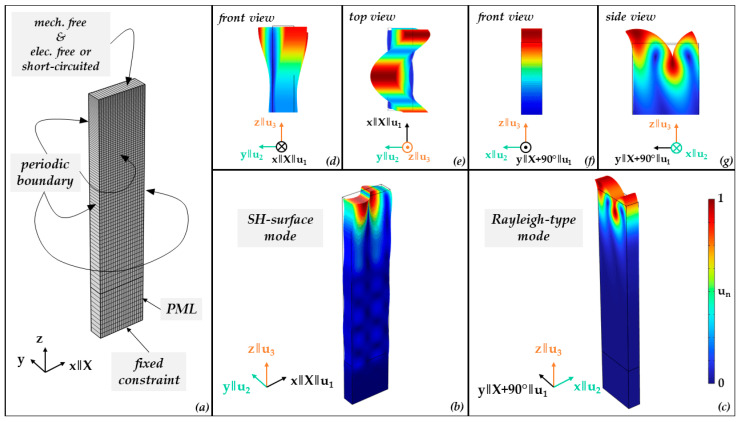
3D FEM eigenfrequency study: (**a**) model of 64LN block with mesh and boundary domain definitions, (**b**) shape of leaky SH-surface mode and (**c**) shape of Rayleigh-type mode. (**d**) Front and (**e**) top view of (**b**); (**f**) front and (**g**) side view of (**c**); color bar corresponds to normalized displacement magnitude $u_{n}=\frac{u_{res}}{u_{res,max}}$ with $u_{res}=\sqrt{u_{1}^{2}+u_{2}^{2}+u_{3}^{2}}$ for all color plots.

Finite-element (FEM) software COMSOL Multiphysics 6.2 is used to perform a modal analysis to search for configurations supporting different modes [15] and, more specifically, where both SH-LSAW and sagittal-polarized wave can be electromechanically excited on the same cut and which directions are beneficial for the excitation of sagittal-polarized wave and of SH-LSAW, respectively. The 3D model (Figure 1) consists of a lithium niobate block of 1 µm in length with 20 mesh elements, exactly one element of 0.3 µm in width and 4 µm in height with 80 elements. The 80 elements have an exponential grow, rate where the lowest element is 5 fold the size of the element at the top. This is set to ensure a finer mesh in the surface region. Surface domains facing length and width directions have periodic boundary conditions. The material constants for lithium niobate are chosen according to [16]; see Table A1. Additionally, a perfectly matched layer (PML) domain of 1 µm height and ten elements in global z direction is used at the bottom to suppress ground reflection of bulk components at the lower boundary with fixed constraint. Crystallographic *X*-axis of lithium niobate corresponds to global x-coordinate and the 64°-rotated Y-cut corresponds to the x-y plane of the model.

**Figure 2:**
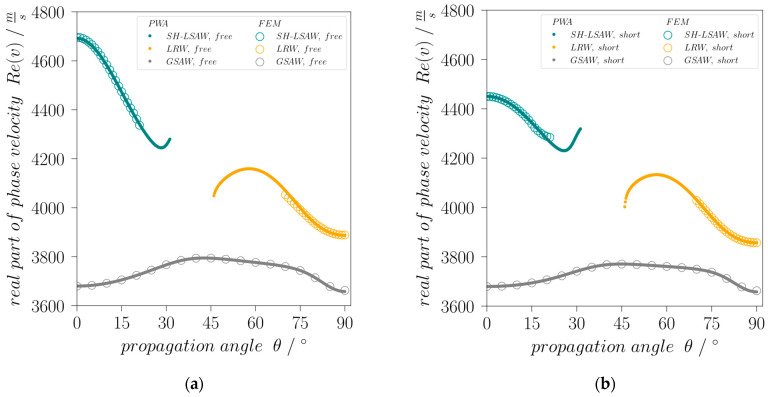
Angular dispersion of phase velocities of SH-LSAW, leaky Rayleigh-type (LRW) wave and generalized SAW (GSAW) branches with (**a**) free and (**b**) short-circuited surface.

An eigenfrequency study with a parametric angular sweep about the global *z*-axis reveals for a rotation of 0°, the existence of an SH eigenmode (Figure 1b) with a complex eigenfrequency of ***f*** = *Re*(***f***) *+ j∙Im*(***f***) *=* (4695.96 *+* 4.5361*j*) MHz, where the very small imaginary component indicates the related acoustic damping caused by leakage into bulk acoustic waves (BAW). This eigenmode corresponds to the well-known shear-horizontal polarized leaky wave (SH-LSAW) propagating along the *X*-axis of 64LN. Considering as wavelength *λ* the spatial periodicity of 1 µm along the length coordinate as defined by the periodic boundary condition and the real part of complex eigenfrequency ***f***, Equation (1) yields the real part of the complex phase velocity ***v*** of the corresponding SH-LSAW mode for free (electrically open) surface, which results in 4695.96 m/s (Figure 2a). This value differs by only 0.084% compared to [14].

(1) $$v=\frac{\lambda }{Re(f)}$$

In addition to the eigenfrequency study, a partial-wave analysis (PWA; see [17] for details) is performed with a custom made software package based on [18] proving the suitability of the FEM model for further investigation.

**Figure 3:**
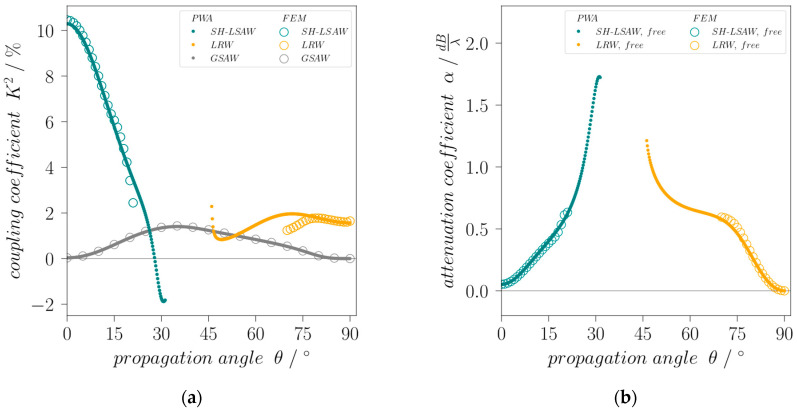
Angular dispersion of (**a**) electromechanical coupling coefficient *K*^2^ for SH-LSAW, leaky Rayleigh-type wave (LRW) and generalized SAW (GSAW) branches and of (**b**) attenuation coefficient *α* for SH-LSAW and LRW for free surface.

In case of the finite-element analysis at a rotation angle of 90°, an eigenmode with ***f*** = (3887.71 + 0*j*) MHz indicates a corresponding SAW mainly polarized within the sagittal plane, i.e., a Rayleigh-type wave (Figure 1c). As this mode is non-leaky only for this special direction but leaky for all other angles, it will be referred to in the following as leaky Rayleigh-type wave (LRW) branch. Additionally, there exists a third, generalized surface wave mode (GSAW) branch with a polarization significantly changing its character over propagation direction and with a phase velocity always lower than SH-LSAW and LRW (Figure 2). To further investigate the possibility to excite the identified SAW modes electrically, the electromechanical coupling coefficient *K*^2^ that is formally defined in terms of the relative phase velocity difference for electrically open and short-circuited surface by Equation (2) [19]

(2) $$K^{2}=\frac{2(v_{free}-v_{short})}{v_{free}}$$

is calculated for the modes as a function of the propagation angle. For this, eigenfrequency analysis and PWA are performed for the whole angular range under the same conditions like before but with an electrically short-circuited substrate surface. *K*^2^ for the leaky SH-SAW at 0° has been calculated to 10.46% which is very close to the value of 11% given in [19]. The leaky Rayleigh-type wave has a *K*^2^ of 1.6% at 90° propagation angle whereas the coupling of the GSAW mode for the angles 0° and 90° is zero (Figure 3a). This means it is practically non-piezoelectric for these directions and can therefore not be excited by electrical fields but by scattering effects [14] making it useless for devices. An interesting effect is also seen in case of the SH-LSAW for angles with high attenuation, where *K*^2^ even reaches negative values when calculated formally using Equation (2) [20]. Note for comparison that commonly used STX-quartz has a *K*^2^ of only 0.12% [19]. Another important feature for the technical deployment of waves is their attenuation, what is especially important for all leaky modes. Figure 3b shows the attenuation coefficient *α_dB/λ_* calculated from complex phase velocity components using Equation (3).

(3) $$\alpha_{dB/\lambda }=10\cdot lg(e)\cdot 4\pi \frac{Im(v)}{Re(v)}\;.$$

For the SH-LSAW propagation along *X*-axis the attenuation has an acceptable minimum of 0.05 *dB*/*λ* whereas for the leaky Rayleigh-type wave (LRW) it is zero for 90° propagation angle *θ*.

## 3. Device Characterization

Based on simulation results, dual-mode delay line devices are prepared on 64°-rotated Y-cut lithium niobate with Euler angles (0°, −26°, 0°) for the SH-SAW and (0°, −26°, 90°) for the attenuation-free Rayleigh wave (RW) as an exceptional case of the LRW branch. The single-side polished piezoelectric substrate has a thickness of 500 µm with the electrode metallization with an overall thickness of 300 nm (295 nm Al on 5 nm Ti) on top. The interdigital transducer (IDT) structures are patterned by mask-less photolithography (MLA 100, Heidelberg Instruments, Heidelberg, Germany) using conventional lift-off technique after e-beam evaporation. All four solid finger IDTs (4 ports, 4P) are of identical design, consisting of 33 finger pairs with a finger width and gap of *λ*/4 *=* 37.5 µm (*λ*_SAW_ = 150 µm) and an aperture of 2 mm. Both delay lines have a length of 50*λ* face-to-face. To reduce coherent reflections at the device edges these are cut at a 30-degree angle to the transducer orientation and covered with highly viscous photoresist. Generally, the radiation of bulk acoustic waves (BAW) into the depth of the substrate as a result of parasitic SH-BAW excitation in the case of Rayleigh wave IDTs as well as the energy leakage of the SH-LSAW can lead to undesired acoustic interference at the surface due to back-reflection from the substrate rear side. To avoid this, a waffle-weave pattern [14] has been cut into the backside of the substrate to diffuse BAW scattering and thereby minimizing negative effects on the sensor signal.

**Figure 4:**
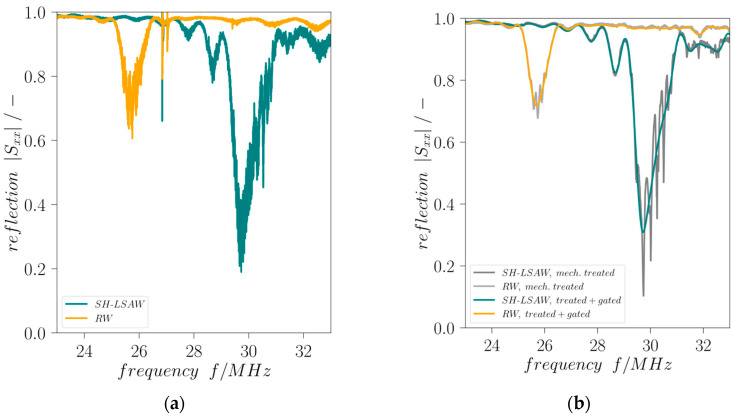
Scattering parameter |*S_xx_*| vs. frequency (**a**) without and (**b**) with mechanical treatment and gating.

All IDTs are electrically pair-wise characterized in respect to their orientation by S-parameter measurements using a vector network analyzer (VNA, E5070B, Keysight Technologies, Santa Rosa, CA, USA). The SAW dual-mode delay line device (DM-DL) is mounted on a custom-made sample holder and electrically connected via gold-plated spring-loaded pins soldered to a printed circuit board (PCB) with conductor-backed coplanar waveguides (CBCPW) of 50 Ω characteristic impedance and SMA connectors. A short-open-load-through (SOLT) calibration is performed at the SMA connector level and the electrical delay caused by the CBCPWs is compensated. For both delay lines, all scattering parameters are measured at the corresponding ports 1, 2 (SH-LSAW) and 3, 4 (RW), i.e., reflection in terms of *S*_11_, *S*_22_, *S*_33_, *S*_44_ and transmission *S*_21_, *S*_12_, *S*_43_, *S*_34_, with all determined at room temperature for unloaded substrate surface. Due to the DM-DL structure, the relations *S_xx_ = S_yy_* and *S_yx_ = S_xy_* are valid for the corresponding ports and are experimentally confirmed. The *|S*_11*,SH-LSAW*_*|* and *|S*_33,*RW*_*|* minimum frequencies are 29.72 MHz and 25.69 MHz, respectively. While the SH-LSAW IDT reflection coefficient shows a minimum of 0.31, the *|S*_33,*RW*_*|* minimum is 0.72 (Figure 4). This is because all IDTs are of identical design and no specific impedance matching was introduced for the Rayleigh wave direction.

**Figure 5:**
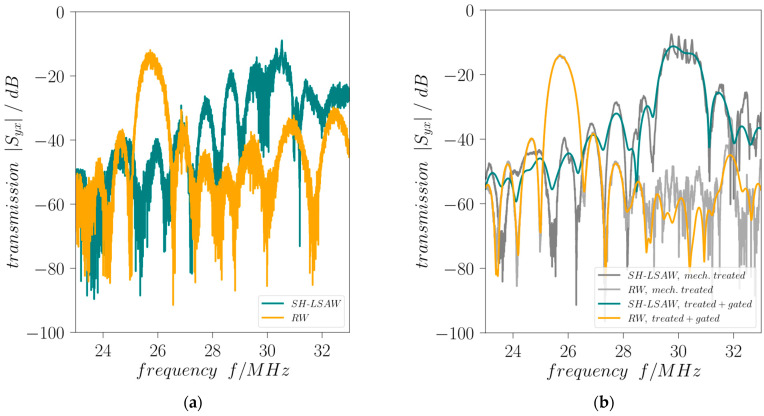
Scattering parameter |*S_yx_*| vs. frequency (**a**) without and (**b**) with mechanical treatment and gating.

Mechanical treatment is applied to the DM-DL devices in order to reduce influences of parasitic BAW contributions as well as distortions caused by edge-reflected surface wave components. Comprising a waffle-weave pattern cut into the chip backside and applying photoresist at the edge-facing end of all IDTs this treatment proves to be effective as the curves of reflection coefficients *|S_xx_|* and transmission coefficients *|S_yx_|* are significantly less distorted (Figure 4 and Figure 5). Moreover, the destructive acoustic interference in the SH-LSAW transmission curve at approximately 30 MHz is also prevented (Figure 5a,b). Further improvement of all S-parameter curves is achieved by signal processing that includes Fourier transformation, application of appropriate gating in the time domain to suppress non-mode-specific contributions and back-transformation into the frequency domain. 

**Figure 6:**
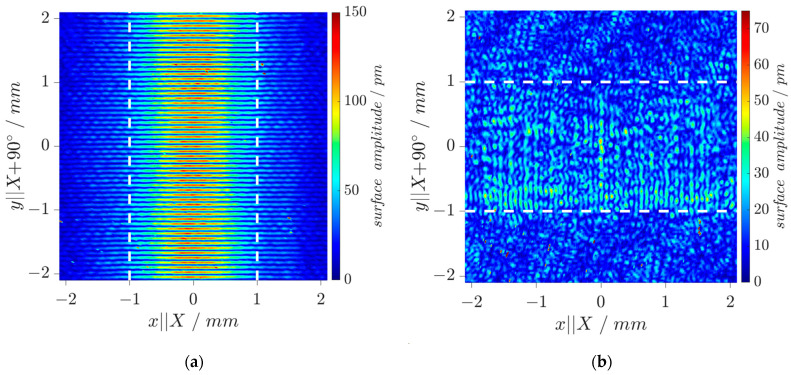
LDV wavefield measurements: Surface displacement amplitudes in surface-normal direction for (**a**) Rayleigh-type wave at 25.69 MHz and (**b**) SH-LSAW at 29.72 MHz (device under test without mechanical treatment; IDT aperture shown by white dashed lines).

The wavefield of an DM-DL device is experimentally investigated by Laser-Doppler-Vibrometry (LDV, UHF-120, Polytec, Waldbronn, Germany) to validate results of the mode simulations. Figure 6a shows measured displacement amplitudes in surface-normal direction (i.e., parallel to global *z*-axis) for a Rayleigh mode pattern along *y‖X*+90° direction at 25.69 MHz with both IDTs activated to check their correct operation. The maximum displacement of the resultant standing wave pattern reaches *u*_3,*max*_ = 120 pm. Figure 6b shows displacement amplitudes for the SH-LSAW at 29.72 MHz under the same operation conditions. As expected from modal analysis, the surface-normal displacements (*u*_3,*max*_ = 37 pm) are much smaller here compared to the Rayleigh mode. Nevertheless, the characteristic standing wave pattern can be clearly identified within the IDT aperture.

## 4. Sensing Experiments

**Figure 7:**
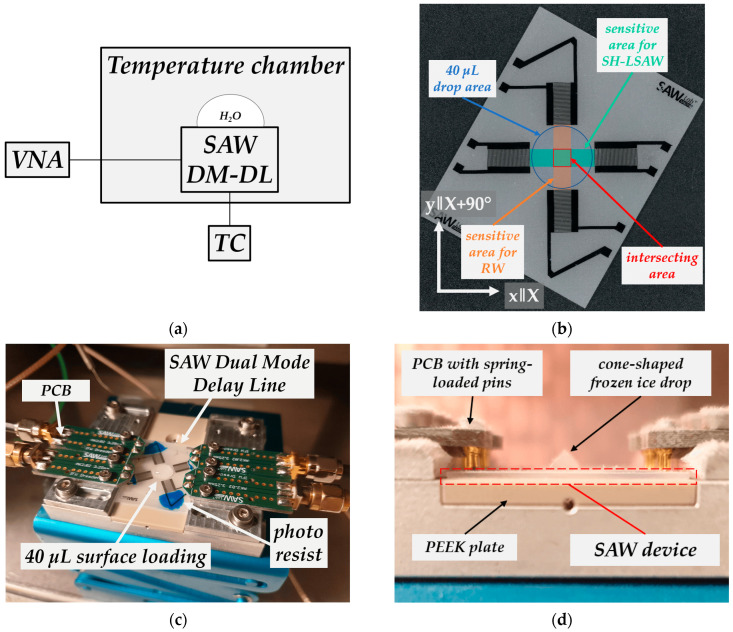
(**a**) Scheme of experimental setup; (**b**) dual-mode delay line device with acoustic area and loaded area; (**c**) device setup inside temperature chamber; (**d**) frozen drop on device.

The experimental test setup to characterize the dual-mode delay line behavior with liquid water and ice loading consists of a temperature chamber (VT 4002, Vötsch, Balingen, Germany) and VNA. Temperature behavior of the DM-DL device within the range of −30 °C to 50 °C is measured in the state without surface loading, resulting in temperature coefficients of frequency (TCF) for both modes. A type-K thermocouple (TC) inserted underneath the custom-made sample holder with its tip directly touching the bottom-side of the piezoelectric chip measures the substrate temperature. Substrate and aluminum sample holder are thermally decoupled by a PEEK plate (Figure 7).

**Figure 8:**
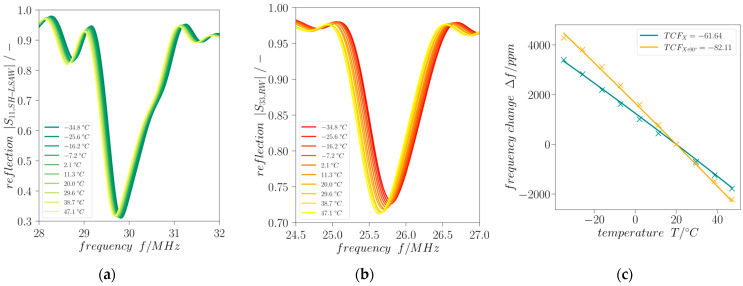
Temperature dependence of time-gated (**a**) |*S_11,SH-LSAW_*| and (**b**) |*S_33,RW_*| within the range −30 °C to 50 °C. (**c**) Derived temperature coefficients of frequency (TCF) for X and X+90° direction.

The frequency shift of the minimum of reflection *|S_xx_|* provides information about the actual device temperature which can be deployed as additional indicator for the presence of icing conditions. Figure 8 shows the according frequency shifts of time-gated reflection curves for SH-LSAW and Rayleigh-type waves (path directions X and X+90°, respectively). There is a linear temperature behavior obtained for both modes in the investigated range of −30 °C to 50 °C yielding temperature coefficients (TCF) calculated from the shift of minimum *|S_xx_|* of *TCF_X_ =* −61.62 ppm/°C and *TCF_X+_*_90*°*_ = −82.11 ppm/°C w.r.t 20 °C as the reference.

**Figure 9:**
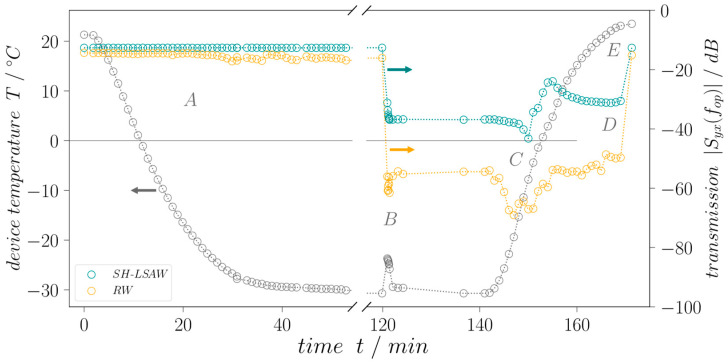
DM-DL device temperature (grey) and time-gated transmission behavior over time for SH-LSAW (green) and RW (yellow) propagation paths. (**A**): cooling in dry conditions, (**B**): water drop application and freezing, (**C**): heating with dew formation and melting, (**D**): water drop completely liquid, and (**E**): dry surface. Dotted lines are for eye guidance only.

To investigate the dual-mode device response on ice loading the transmission behavior of both propagation paths is measured. Figure 9 shows the temperature of the DM-DL device measured underneath the piezoelectric substrate during the experiment (grey curve) as well as the regarding time-gated transmission coefficients *|S_yx_* (*f_op_*)*|* at operating frequency *f_op_* defined by the center frequency between the zeros next to the transmission main lobe. While the device is still in dry conditions, without any surface load the temperature chamber is cooled down to −30 °C until the piezoelectric substrate temperature stabilizes as can be concluded from stable S-parameters. The cooling takes approximately 40 min. After 120 min, the chamber door is opened to apply a drop of tap water (non-deionized, room temperature) with a volume of 40 µL by pipette (Eppendorf Research, Hamburg, Germany) onto the sensitive surface of the sensor device including both wave propagation paths (Figure 7b,c). Considered the device geometry, a 40 µL drop ensures complete wetting of the whole sensitive area. The chamber door is closed immediately after the deposition. Due to the difference between water and the DM-DL substrate temperature, a rise of 7.0 °C quickly reverting to approximately −30 °C is measured, and is indicated in Figure 9 by a small peak. During the droplet application, comparatively warm and humid air streams into the chamber, leading to a deposition of a thin and weakly adherent ice layer on all parts with a sub-freezing temperature including the SAW device. Here, a small decrease in transmission for the Rayleigh wave can be seen as the thin ice layer covers the full device including IDTs and attenuates the sagittal polarized Rayleigh wave there. The shear-horizontal polarized wave is not influenced due to the very low adhesion of the ice to the sensitive surface. As the air inside the chamber cools down again quickly and becomes cooler than the device setup the thin ice layer vanishes due to sublimation. The applied liquid water load freezes in an approximate time frame of less than 5 min, resulting in a cone-shaped ice tip (Figure 7d). Underlaying this procedure is the assumption that the surface area covered by ice is equal to that formerly covered by the liquid water. Moreover, thermal expansion of the water as well as changes in the substrate surface energy during the freezing process are neglected.

A waiting time of 20 min after water drop application is held to ensure stable S-parameter until the heating to room temperature is initialized. The increase in air temperature inside the chamber leads to dew formation on all surfaces which include the DM-DL device transducers, leading to additional attenuation while the water droplet is still mostly frozen. As soon as the device temperature reaches 15 °C, the dew vaporized again clearing the IDTs from any liquid. After the heating period to room temperature (23 °C) of approximately 30 min, the device sensitive area is loaded with melted water only. A final S-parameter measurement is performed after completely removing the liquid water to ensure same transmission values before and after the experiment.

**Figure 10:**
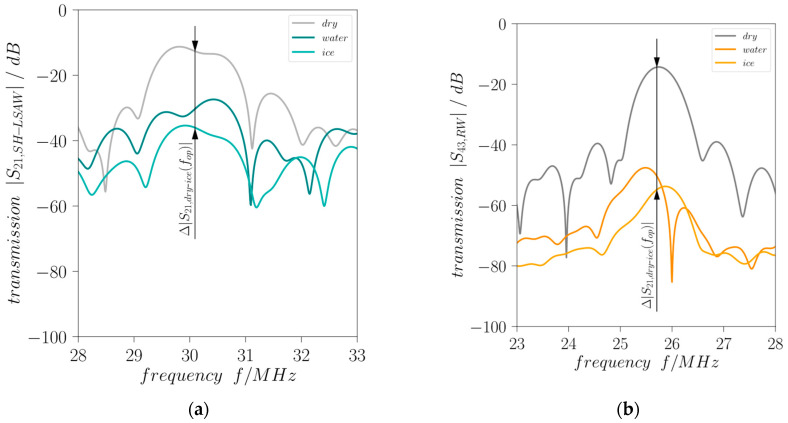
Effect of surface loading on DM-DL transmission characteristics (**a**) *|S*_21,*SH-LSAW*_*|* and (**b**) *|S*_43,*RW*_*|* (dry: no surface load, water: 40 µL water with complete wetting (both at 23 °C); ice: 40 µL ice (−30 °C); all curves are time-gated).

**Figure 11:**
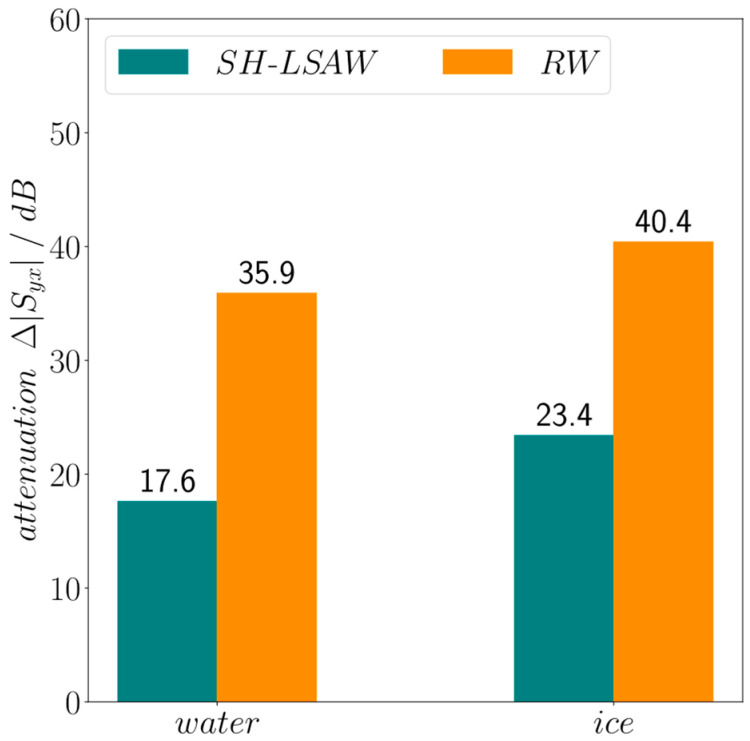
Increase in SAW attenuation ∆*|S*_21,*mode*_*|* dependent on mode and surface loading for the DM-DL operating frequencies indicated in Figure 10 w.r.t. unloaded surface as reference.

Figure 10 illustrates the measured transmission curves at the beginning (*t* = 0) of the experiment without surface load (dry), at approximately 137 min with ice loading and at end just before drying the surface with liquid water loading. Both acoustic modes propagating in perpendicular directions on the DM-DL device experience a different degree of attenuation in response to surface loading by liquid water which leads to a decrease in the corresponding transmission curves. Moreover, for both modes freezing of the liquid leads to a further decrease in the transmission. Important to mention, an ideal SAW attenuation is indicated by a nearly constant decrease in transmission over the whole frequency range whereas here also (changing) dielectric properties of the water drop loading will influence the characteristics [21]. As expected, the shear-horizontal polarized wave shows a lower attenuation compared to the Rayleigh-type wave. This is due to the neglectable out-of-plane displacement of an ideal SH-LSAW at the substrate surface, where the Rayleigh wave has its maximum surface-normal particle displacement, leading to strong acoustic leakage by radiation of longitudinal BAW into the surface load for the latter case. Moreover, an SH-LSAW shows only weak shear coupling to liquid loads like water with a low dynamic viscosity of approximately 1 mPas at room temperature [22]. Nevertheless, in the device under test the SH-LSAW experiences acoustic leakage as the real wavefield shows also minor particle displacement in surface-normal direction as revealed by LDV measurement (Figure 6b). This non-ideal behavior leads to a mechanical coupling to the liquid water load, which results in the decrease in *|S*_21,*SH-LSAW*_*|*. With increasing viscosity during the freezing process also the applied shear forces lead to shear stress at the interface between load and substrate surface what results in a further decrease in the transmission coefficient. The sagittal polarized Rayleigh-type wave with its major particle displacement in surface-normal direction witnesses a high attenuation *|S*_43,*RW*_*|* under liquid water loading right away which even increases during the freezing process. A quantitative view on the load-dependent increase in attenuation for both modes provides Figure 11. At the DM-DL operating frequencies defined by the center frequency between the zeros next to the transmission main lobe a dramatic increase in almost 36 dB in Rayleigh-mode attenuation is present for water loading, further increasing by 5.5 dB when the water freezes. In contrast, SH-LSAW experiences generally a much lower increase in attenuation due to surface loading with differences of ~18 dB for liquid water and ~23 dB when it freezes.

## 5. Conclusions

The discussed results show that it is possible to electrically excite Rayleigh-type waves on 64°-rotated Y-cut lithium niobate, additionally to the commonly used shear-horizontal polarized wave, at a propagation angle of 90° relative to the *X* direction. These findings allow the fabrication of a 4-port dual-mode delay line device. A waffle-weave patterned cut on the device backside to minimize leaking bulk wave interferences as well as the application of photoresist as an acoustic absorber removed noise from the measured S-parameters and eased the evaluations. The 4-port arrangement allowed the determination of reflection coefficients as well as transmission coefficients. While the IDT reflection coefficient *|S_xx_|* remain unaffected by surface loading of the sensitive area within the propagation path), it delivers important information on the real device temperature. Additionally, the different change in transmission characteristics *|S_yx_|* for both acoustic modes in response on surface loading allow the precise differentiation of liquid water and ice load. In combining both effects, the presented DM-DL device demonstrates its potential for a sensor to simultaneously determine surface load condition and temperature for reliable ice detection on industrial surfaces.

## Data Availability

Data are contained within the article.

## Appendix A

**Table A1:** Material constants for LiNbO_3_ at room temperature (T = 26 °C) from [16].

Elastic Constants in 10^10^ N/m^2^	
$c_{11}^{E}$	19.839 ± 0.089
$c_{12}^{E}$	5.472 ± 0.097
$c_{13}^{E}$	6.513 ± 0.193
$c_{14}^{E}$	0.788 ± 0.004
$c_{33}^{E}$	22.790 ± 0.324
$c_{44}^{E}$	5.965 ± 0.008
Piezoelectric constants in C/m^2^	
$e_{15}$	3.69 ± 0.06
$e_{22}$	2.42 ± 0.04
$e_{31}$	0.30 ± 0.08
$e_{33}$	1.77 ± 0.12
Dielectric constants in ε_0_	
$\varepsilon_{11}^{S}$	45.6 ± 1.5
$\varepsilon_{33}^{S}$	26.3 ± 1.6
Mass density in kg/m^3^	
ρ	4628
